# Ensemble-Based Mutational Profiling and Network Analysis of the SARS-CoV-2 Spike Omicron XBB Lineages for Interactions with the ACE2 Receptor and Antibodies: Cooperation of Binding Hotspots in Mediating Epistatic Couplings Underlies Binding Mechanism and Immune Escape

**DOI:** 10.3390/ijms25084281

**Published:** 2024-04-12

**Authors:** Nishank Raisinghani, Mohammed Alshahrani, Grace Gupta, Gennady Verkhivker

**Affiliations:** 1 Keck Center for Science and Engineering, Graduate Program in Computational and Data Sciences, Schmid College of Science and Technology, Chapman University, Orange, CA 92866, USA; rai.r.nick@gmail.com (N.R.); alshahrani@chapman.edu (M.A.); grgupta@chapman.edu (G.G.); 2 Department of Biomedical and Pharmaceutical Sciences, Chapman University School of Pharmacy, Irvine, CA 92618, USA

**Keywords:** SARS-CoV-2 spike protein, ACE2 host receptor, mutational scanning, binding energetics, antibody binding, immune evasion, allosteric communications

## Abstract

In this study, we performed a computational study of binding mechanisms for the SARS-CoV-2 spike Omicron XBB lineages with the host cell receptor ACE2 and a panel of diverse class one antibodies. The central objective of this investigation was to examine the molecular factors underlying epistatic couplings among convergent evolution hotspots that enable optimal balancing of ACE2 binding and antibody evasion for Omicron variants BA.1, BA2, BA.3, BA.4/BA.5, BQ.1.1, XBB.1, XBB.1.5, and XBB.1.5 + L455F/F456L. By combining evolutionary analysis, molecular dynamics simulations, and ensemble-based mutational scanning of spike protein residues in complexes with ACE2, we identified structural stability and binding affinity hotspots that are consistent with the results of biochemical studies. In agreement with the results of deep mutational scanning experiments, our quantitative analysis correctly reproduced strong and variant-specific epistatic effects in the XBB.1.5 and BA.2 variants. It was shown that Y453W and F456L mutations can enhance ACE2 binding when coupled with Q493 in XBB.1.5, while these mutations become destabilized when coupled with the R493 position in the BA.2 variant. The results provided a molecular rationale of the epistatic mechanism in Omicron variants, showing a central role of the Q493/R493 hotspot in modulating epistatic couplings between convergent mutational sites L455F and F456L in XBB lineages. The results of mutational scanning and binding analysis of the Omicron XBB spike variants with ACE2 receptors and a panel of class one antibodies provide a quantitative rationale for the experimental evidence that epistatic interactions of the physically proximal binding hotspots Y501, R498, Q493, L455F, and F456L can determine strong ACE2 binding, while convergent mutational sites F456L and F486P are instrumental in mediating broad antibody resistance. The study supports a mechanism in which the impact on ACE2 binding affinity is mediated through a small group of universal binding hotspots, while the effect of immune evasion could be more variant-dependent and modulated by convergent mutational sites in the conformationally adaptable spike regions.

## 1. Introduction

The wealth of structural and biochemical investigations conducted on the spike (S) glycoprotein of the SARS-CoV-2 virus have provided crucial insights into the mechanisms that regulate virus transmission and immune evasion. This glycoprotein, tasked with facilitating the virus’s entry into host cells, undergoes significant conformational shifts between closed and open states. These structural transitions are driven by allosterically coupled motions of the flexible N-terminal S1 subunit, which includes the N-terminal domain (NTD), the receptor-binding domain (RBD), and two structurally conserved subdomains known as SD1 and SD2 [1,2,3,4,5,6,7,8,9]. The intricate coordination among these structural domains orchestrates conformational shifts within the S protein, fluctuating between the closed state with the RBD down, and the open state with the RBD up. The efficacy of the S protein in recognizing and binding to host cell receptors, as well as its ability to evade immune detection, relies on its capacity to navigate between these distinct structural states, where the dynamic equilibrium and thermodynamic preferences towards particular functional form can be modulated by the binding partners [10,11,12,13,14,15]. The extensive insights gained from biophysical studies have improved our comprehension of the S protein trimer, illuminating the intricate interplay between thermodynamics and kinetics that govern spike mechanisms. These investigations have revealed a nuanced understanding of how mutations and long-range interactions, particularly between the dynamic S1 subunit and the more rigid S2 subunit, determine structural alterations within the S protein trimer and influence population shifts between the open and closed RBD states [16,17,18].

The abundance of cryo-electron microscopy (cryo-EM) and X-ray structures pertaining to the S protein variants of concern (VOCs) has deepened our insight into the evolutionary adaptability of the S protein and the range of molecular mechanisms through which viral fitness is determined. These mechanisms are mediated by a complex balance between binding with the ACE2 host receptor and the efficiency of the immune escape [19,20,21,22,23,24,25,26,27,28]. The most recent cryo-EM structures and biochemical analyses of the S trimers across the BA.1, BA.2, BA.3, and BA.4/BA.5 Omicron variants have revealed a significant reduction in binding affinity for the BA.4/BA.5 subvariants. This confirms that BA.2 exhibits higher binding affinities compared to the other Omicron variants [29,30]. Structural and biophysical examinations of the Omicron BA.2.75 variant have indicated that, under neutral pH conditions, the BA.2.75 S-trimer exhibits the highest thermal stability among the Omicron variants, ranking above BA.1, BA.2.12.1, BA.5, and BA.2. Surface plasmon resonance (SPR) experiments were conducted for multiple Omicron subvariants, including BA.1, BA.2, BA.3, BA.4/5, BA.2.12.1, and BA.2.75, revealing a notably higher ACE2 binding affinity in the BA.2.75 subvariant [31,32,33]. Structure–functional investigations have confirmed that the BA.2.75 variant can be endowed with significant antibody evasion potential while featuring enhanced ACE2 binding as well as improved growth efficiency and intrinsic pathogenicity [33].

The appearance of the XBB.1 subvariant within the Omicron lineage serves as a noteworthy illustration of viral evolution. XBB.1.5 bears a notable resemblance to XBB.1, and is distinguished by a singular and rare two-nucleotide substitution in the RBD when compared to the ancestral strain [34]. Biophysical investigations of the S trimer binding with ACE2 have revealed that the binding affinities of BQ.1 and BQ.1.1 are comparable to that of the BA.4/BA5 variants. In contrast, XBB and XBB.1 exhibit binding affinities similar to the BA.2 variant. Biochemical studies demonstrated that monoclonal antibodies, which were effective against the original Omicron variant, have exhibited reduced efficacy against the XBB.1 and BQ.1 subvariants [35]. The exceptional binding affinity of the XBB.1.5 RBD to ACE2 has been confirmed in other investigations, also revealing comparable antibody evasion for XBB.1 and XBB.1.5, with much greater transmissibility for the XBB.1.5 variant [36,37]. Cryo-EM analysis of the XBB.1.5 S ectodomain has highlighted a higher ACE2 binding affinity compared to XBB.1, which may contribute to the observed growth advantages and increased transmissibility of the XBB.1.5 variant [38]. The XBB sublineages XBB.1.5 and XBB.1.16, which share an F486P substitution, have become predominant worldwide (https://nextstrain.org/ (accessed on 5 February 2024)) [39]. XBB.1.16 emerged independently from XBB.1.5 and features two additional substitutions (E180V in the NTD and T478R in the RBD), showing a greater growth advantage [40]. Emerging variants display increased infectivity and transmissibility compared to previous Omicron variants, and some RBD residues (R346, K356, K444, V445, G446, N450, L452, N460, F486, F490, R493, and S494) have shown mutation in at least five new independent Omicron lineages.

The XBB descendants that bear an additional F456L mutation, including EG.5 and EG.5.1 (XBB.1.9.2.5.1), have become one of the currently predominant lineages circulating worldwide [41]. EG.5 harbors only one additional F456L substitution relative to XBB.1.5, while its immediate descendant EG.5.1 features Q52H in the NTD and F456L in the RBD [41]. Biochemical studies have shown that the ACE2 binding of EG.5.1 RBD is weaker compared to that of XBB.1.5, but EG.5.1 exhibits significantly greater immune resistance. F456L substitution has been implicated as an important determinant in enhanced immunological potential [41]. EG.5 and EG.5.1 were found to be moderately more resistant for class one monoclonal antibodies as compared to XBB.1.5; this is largely mediated by a single F456L mutation on the RBD [42]. This observation was also confirmed in studies of immune evasion of the EG.5.1 subvariant, which showed that F456L mutation, rather than Q52H, drives the enhanced neutralization escape of EG.5.1 [43]. EG.5.1 has further evolved, resulting in a descendant lineage named HK.3 (XBB.1.9.2.5.1.1.3), which harbors L455F (EG.5.1 + L455F) [44]. XBB subvariants bearing a combination of L455F and F456L flipped substitutions are termed “FLip” variants. These subvariants include JG.3 (XBB.1.9.2.5.1.3.3), JF.1 (XBB.1.16.6.1), GK.3 (XBB.1.5.70.3), and JD.1.1, all of which emerged convergently, indicating that acquisition of the L455F/F456L “combo” can confer a growth advantage to XBB in the human population [44,45].

Biochemical studies of ACE2 binding with XBB.1.5, XBB.1.5 + L455F, XBB.1.5 + F456L, and XBB.1.5 + L455F + F456L have revealed that L455F reduces ACE2 affinity, while the adjacent residue flipping of L455F and F456L leads to enhanced ACE2 binding affinity and also induces an enhanced immune escape in the class one monoclonal antibodies [46]. The Omicron subvariant BA.2.86, derived from the BA.2 variant, exhibits significant genetic differences compared to its predecessors [47,48,49,50,51]. Biophysical studies have measured ACE2 binding affinities, showing that the XBB.1.5 and EG.5.1 spikes exhibit comparable affinities to ACE2, while two different constructs of the BA.2.86 S protein showed a >2-fold increase in binding affinity [48]. The immune evasion capability of the BA.2.86 subvariant was evaluated using a panel of XBB.1.5-effective neutralizing antibodies, revealing that BA.2.86 is antigenically distinct from XBB.1.5 and can escape XBB-induced neutralizing antibodies [49]. A comparative functional analysis of immune evasion and infectivity for BA.2.86, EG.5.1, and FLip variants showed that BA.2.86 is less resistant to neutralization compared to XBB.1.5, EG.5.1, and FLip variants [52]. Notably, the FLip variant exhibited stronger immune escape than its parental variant XBB.1.5 due to both L455F and F456L mutations. JN.1 is a variant of BA.2.86 which emerged independently from Omicron BA.2 and harbors an additional L455S mutation, which confers enhanced immune escape capability [53]. A comparative biochemical analysis showed a reduction in ACE2 binding affinity for JN.1, indicating that its enhanced immune evasion capabilities come at the expense of reduced ACE2 binding [53]. The evolutionary pattern of XBB.1.5, E.5.1, and FLip variants suggests a predominant role of immune evasion, where compensatory mutations L455GF/F456L may have emerged to restore the decreased ACE2 affinity.

Deep mutational scanning (DMS) experiments and functional studies have suggested that evolutionary windows for the Omicron variants could be enhanced through epistatic interactions between variant mutations, in which immune escape mutations can individually reduce ACE2 binding, but are compensated for through epistatic couplings with affinity-enhancing Q498R and N501Y mutations [54,55,56,57,58]. Recent evolutionary studies have revealed strong epistasis between pre-existing substitutions in BA.1/BA.2 variants and antibody resistance mutations acquired during selection experiments, suggesting that epistasis can also lower the genetic barrier for antibody escape [59]. DMS analysis of the XBB.1.5 and BA.2 S proteins showed that the strongest escape mutations are in the RBD sites 357, 420, 440, 456, and 473, but escape mutations can also reside outside the RBD, with many of them decreasing ACE2 binding [60]. Another study examined the preference of each RBD mutation on antibody escape and human ACE2 binding using BA.5-based DMS profiles and revealed the most important functional hotspots at R403S/K, N405K, N417Y, Y453S/C/F, L455W/F/S, F456C/V/L, and H505Y/D positions [61]. Recent DMS experiments examining BQ.1.1 and XBB.1.5 RBDs binding with ACE2 have unveiled the important role of epistatic couplings between R493Q and the mutations at positions Y453, L455, and F456 that define the EG.5.1 and FLip lineages [62]. Convergent evolution of the XBB lineages has shown that the coordination of evolutionary paths at different sites may be largely due to epistatic, rather than random, selection of mutations [63,64].

Computer simulation studies have provided important atomistic insights into understanding the dynamics of the SARS-CoV-2 S protein and the effects of Omicron mutations [65,66,67,68,69,70]. The conformational dynamics and allosteric modulation of SARS-CoV-2 S was studied using an smFRET imaging assay, showing the presence of long-range allosteric control of the RBD equilibrium and antibody binding [71]. The effects of SARS-CoV-2 mutations in the stable fusion core of the S protein on the pre-fusion and post-fusion structures were investigated in long molecular dynamics (MD) simulations, revealing changes in the inter-monomer salt bridge in the post-fusion conformation that can affect protein flexibility and lead to reduced compactness in the assembly [72]. Integrative computational modeling approaches have revealed that the S protein could function as allosteric regulatory machinery [73,74,75,76,77]. By combining atomistic simulations and a community-based network model of epistatic couplings, we found that convergent Omicron mutations such as G446S (BA.2.75, BA.2.75.2, XBB), F486V (BA.4, BA.5, BQ.1, BQ.1.1), F486S, F490S (XBB.1), and F486P (XBB.1.5) can display epistatic relationships with the major stability and binding affinity hotspots [75]. MD simulations and Markov state models characterized conformational landscapes of the XBB.1, XBB.1.5, BQ.1, and BQ.1.1 Omicron variants and their complexes, showing that convergent mutation sites could modulate conformational plasticity in the flexible adaptable regions [77]. Recent computational studies have suggested that Omicron mutations have variant-specific effects on conformational changes in the S protein, leading to the formation and evolution of druggable cryptic pockets [78,79,80].

In this study, we performed a computational study of binding energetics for a spectrum of Omicron variants including the BA.1, BA2, BA.3, BA.4/BA.5, BQ.1.1, XBB.1, XBB.1.5, and XBB.1.5 + L455F/F456L variants. By combining evolutionary analysis, MD simulations, and ensemble-based mutational scanning of the S-RBD residues in their complexes with ACE2, we identified structural stability and binding affinity hotspots. In agreement with the results of deep mutational scanning experiments, our quantitative analysis correctly reproduced strong and variant-specific epistatic effects in the XBB.1.5 and BA.2 genetic variants, showing a central role of the Q493/R493 hotspot in modulating epistatic couplings between the convergent mutational sites L455F and F456L. We also conducted structure-based mutational analysis of the S protein binding with different classes of RBD-targeted antibodies, focusing specifically on the role of XBB.1.5 mutations, as well as L455F, F456L, and FLip mutations, in mediating resistance to class one antibodies. We presented evidence of inter-dependence between binding affinity hotspots and antibody resistance substitutions, which is controlled by the epistatic couplings of Y501, R498, Q493, L455 and F456 residues. This study provides a quantitative rationale for a mechanism in which the impact on ACE2 binding affinity is mediated through a small group of universal binding hotspots, while the effect of immune evasion could be more variant-dependent and modulated by convergent mutational sites in the conformationally adaptable spike regions.

## 2. Results and Discussion

### 2.1. Evolutionary and Phylogenetic Analysis of Differences between BA.2 and XBB.1.5 Lineages

**Figure 1:**
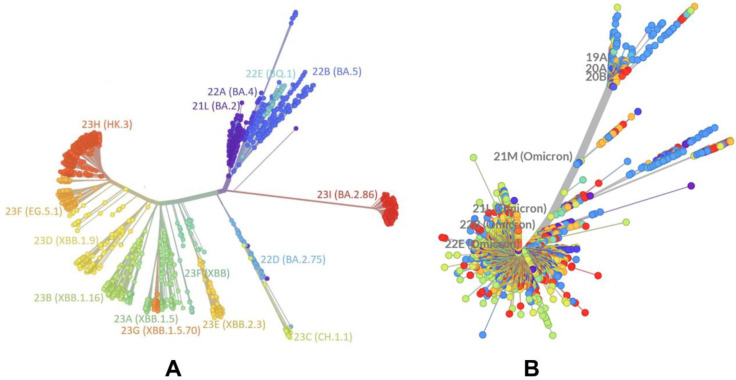
An overview of the phylogenetic analysis and divergence of Omicron variants including XBB variants 23A (XBB.1.5) and 23G (XBB.1.5.70). The graph was generated using Nextstrain, an open-source project for real time tracking of evolving pathogen populations (https://nextstrain.org/). (**A**) The phylogenetic analysis focused on BA.2 and XBB lineages. (**B**) A global phylogenetic view of Omicron variants. All colors for Omicron variant clades on the panels (**A**,**B**) are generated automatically by the Nextstrain program (https://nextstrain.org/).

The evolutionary differences and divergence of XBB linages among Omicron variants are illustrated by the phylogenetic analysis of XBB variants using their corresponding clades nomenclature from Nextstrain, an open-source project for real-time tracking of evolving pathogen populations (https://nextstrain.org/) [39]. Nextstrain provides dynamic and interactive visualizations of the phylogenetic tree of SARS-CoV-2, allowing users to explore the evolutionary relationships between different lineages and variants. This approach assigns SARS-CoV-2 variants a clade when they reach a frequency of 20% globally at any time point. A new clade should be at least two mutations away from its parent major clade. According to the Nextstrain-based evolutionary analysis (Figure 1, Figures S1 and S2), the XBB.1 subvariant is a recombinant of lineage BJ.1 (BA.2.10.1.1) and BM.1.1.1 (BA.2.75.3.1.1.1), with a breakpoint in the S1 region of the spike subunit XBB (has the mutations S:V445P (from BJ.1) and S:N460K (from BM.1.1.1), which are unique to each parent. XBB.1 has NTD mutations V83A, H146Q, Q183E, V213E, and G252V, and specific RBD mutations (G339H, R346T, L368I, V445P, G446S, N460K, F486S, F490S, and reversed R493Q) (Table 1). XBB.1.5 (23A clade) is a recombinant variant, as it descends from XBB (22F clade). XBB.1.5 has additional S mutations, S:G252V and S:S486P, that are also shared by XBB.1.5 + F456L (EG.5) and XBB.1.5 + L455F/F456L (XBB.1.5.70).

**Figure 2:**
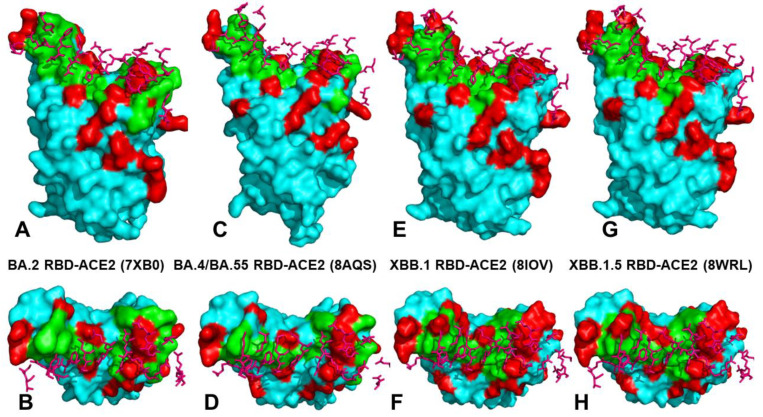
Structural organization and binding epitopes of the SARS-CoV-2-RBD Omicron complexes with human ACE enzyme. (**A**) The cryo-EM structure of the BA.2 RBD -ACE2 complex, pdb id 7XB0 (only RBD is shown). The RBD is in cyan surface, the binding epitope is in green surface, and the BA.2 RBD mutations are in red. (**B**) The top view of the BA.2 RBD. The ACE2 binding residues are shown in pink sticks. (**C**) The cryo-EM structure of the BA.4/BA.5 RBD -ACE2 complex, pdb id 8AQS (only RBD is shown). The RBD is in cyan surface, the binding epitope is in green surface, the BA.4/BA.5 RBD mutations are in red. (**D**) The top view of the BA.4/BA5 RBD. The ACE2 binding residues are shown in pink sticks. (**E**) The cryo-EM structure of the XBB.1 RBD -ACE2 complex, pdb id 8IOV (only RBD is shown). The RBD is in cyan surface, the binding epitope is in green surface, the XBB.1 RBD mutations are in red. (**F**) The top view of the XBB.1 RBD with the ACE2 binding residues shown in pink sticks. (**G**) The cryo-EM structure of the XBB.1.5 RBD -ACE2 complex, pdb id 8WRL (only RBD is shown). The RBD is in cyan surface, the binding epitope is in green surface, the XBB.1.5 RBD mutations are in red. (**H**) The top view of the XBB.1.5 RBD with the ACE2 binding residues in pink sticks.

The mutational landscape of XBB variants shows that these variants are highly similar (Table 1). XBB.1.5.70 (23G clade) descended from the variant XBB.1.5, and has the ‘FLip’ genotype (S:L455F and S:F456L). It emerged in early 2023 in South America, becoming dominant in Brazil around September 2023. In the second half of 2023, it was responsible for more than a third of global XBB.1.5-derived sequences. In sequences collected since 1 September 2023, it has been most common in South America, in particular Brazil (48%), Chile (27%), Argentina (23%), and Colombia (12%). Outside of South America, it has been most common in Japan (8%) and Italy (5%), and has been present at rates of 1–5% in North America and Europe (https://nextstrain.org/). [39]. Phylogenomic reconstruction indicates that the genomes of BQ.1 (clade 22E) are clustered within the not-monophyletic GSAID Clade 21L, with a close relationship with the BA.5 Omicron subvariant (Figure 1). BQ.1, which is a direct descendant of BA.5, has additional spike mutations in some key antigenic sites (K444T and N460K). Its first descendant, BQ.1.1, carries a further additional mutation R346T [81,82]. Despite considerable mutational differences between newly emerged Omicron variants, structural analysis of the RBD complexes with ACE2 for these variants has revealed highly similar RBD conformations and the same binding mode of interactions, rendering overall very minor differences in the crystallographic conformations (Figure 2 and Figure S3).

### 2.2. Mutational Profiling Analysis of Omicron Variants Identifies Key Identifies Universal and Variant-Specific Structural Stability and Binding Affinity Hotspots in the SARS-CoV-2 RBD Complexes with ACE2

We performed MD simulation studies of the RBD–ACE2 complexes for the BA.1-BA.4/BA.5, BQ.1.1, and XBB variants (Supplementary Materials, Figures S4 and S5). The RMSD profiles for the RBD residues showed convergence of the MD trajectories for the BA.2 and XBB.1.5 complexes, where all three trajectories reached a steady equilibrium state after 400 ns (Supplementary Materials, Figure S4). The divergence of the RMSD profiles for the XBB.1 complex (Supplementary Materials, Figure S4) suggested a more heterogeneous ensemble and a greater flexibility of ACE2. The RMSD profiles for the Omicron BQ.1.1 RBD showed convergence for all microsecond trajectories, reaching the steady state after ~300 ns (Supplementary Materials, Figure S5). The RMSDs for the ACE2 displayed a certain degree of variability among the trajectories of the BQ.1 and BQ.1.1 variants, indicating functionally significant plasticity of both binding partners in the RBD–ACE2 complexes. In general, the MD simulations suggested that the RBD residues in the XBB.1.5 and BQ.1.1 complexes undergo relatively moderate fluctuations as compared to the more dynamic parent variants XBB.1 and BQ.1. Although the conformational dynamics of the Omicron RBD–ACE2 complexes for all studied variants are generally similar, some variants such as XBB.1.5 and BQ.1.1 appeared to induce greater stability of the RBD residues and RBD–ACE2 interfaces (Supplementary Materials, Figures S4 and S5).

**Figure 3:**
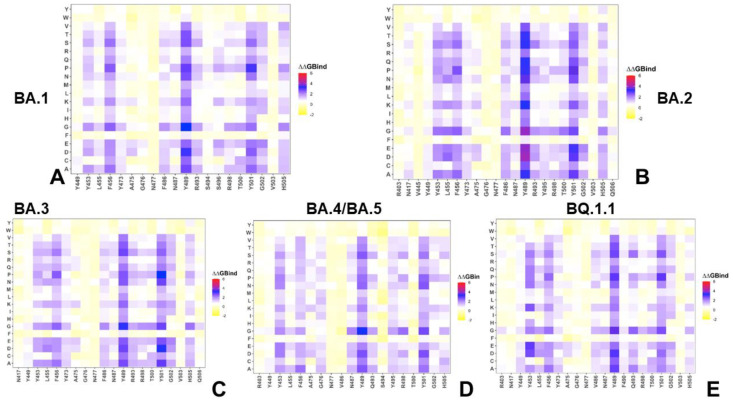
Mutational profiling of the RBD intermolecular interfaces in Omicron RBD–ACE2 complexes. The mutational scanning heatmaps are shown for the interfacial RBD residues in the BA.1 RBD–ACE2 complex (pdb id 7WBP) (**A**), BA.2 RBD–ACE2 complex (pdb id 7XB0) (**B**), BA.3 RBD–ACE2 complex (pdb id 7XB1) (**C**), BA.4/BA.5 RBD–ACE2 complex (pdb id 8AQS) (**D**) and BQ.1.1 RBD–ACE2 complex (pdb id 8IF2) (**E**). The standard errors of the mean for binding free energy changes and are within ~0.11–0.15 kcal/mol, using averages based on a total of 1000 samples obtained from the three MD trajectories for each complex.

Using conformational ensembles obtained from MD simulations, we performed a systematic mutational scanning of the RBD residues in RBD–ACE2 complexes (Figure 3). In silico mutational scanning was performed by averaging the binding free energy changes over the equilibrium ensembles, allowing for predictions of the mutation-induced changes of the binding interactions and the stability of the complex. The resulting mutational scanning heatmaps were reported for the RBD binding interface residues that made stable contacts with ACE2 in the course of simulations. To provide a systematic comparison, we constructed mutational heatmaps for the RBD interface residues of the BA.1 (Figure 3A), BA.2 RBD–ACE2 (Figure 3B), BA.3 (Figure 3C), and BA.4/BA.5 RBD–ACE2 complexes (Figure 3D), and the BQ.1.1 complex (Figure 3E). Consistent with DMS experiments on SARS-CoV-2 S VOCs [54,55,56,57,58,59,60], the hydrophobic residues Y453, L455, F456, F486, Y489, and Y501 were shown to play a decisive role in the stability and binding of all Omicron variants. The large destabilization changes were more pronounced for Y453, L455, and F456, while also showing high sensitivity for F486, N487, R493, T500, Y501, and H505 residues on ACE2 binding (Figure 3A–C). This analysis was also consistent with our previous studies, suggesting that these conserved hydrophobic RBD residues may be universally important for RBD stability and binding across all Omicron variants [83,84]. The common energetic hotspots Y453, F456, Y489, and Y501 also emerged as critical stability and binding hotspots in the experimental DMS studies [54,55,56]. The mutational scanning heatmap for the S Omicron RBD residues shows that the largest and most consistent destabilization changes were observed for the Y489 and Y501 residues (Figure 3). Strikingly, all modifications in these positions resulted in large losses of stability and binding affinity. Another group of the interfacial RBD residues that were shown to make critical contributions to protein stability were at the Y453, L455, and F456 positions. Mutations in these positions showed a consistent destabilization pattern, especially in the BA.2 (Figure 3B) and BA.3 variants (Figure 3C). Our results also confirmed that the Y501 position is the most critical binding affinity hotspot in the S Omicron RBD complex with ACE2. This is consistent with the experimental data, showing that N501Y mutation alone can induce a six-fold improvement in binding affinity [85]. Moreover, a tight cluster of binding affinity hotspots in this region, formed by Y501, R498, S496, and H505 residues, make the dominant contribution to the binding affinity (Figure 3). Although structural and dynamic similarities yield fairly similar energetic heatmaps, there are a number of notable differences that provide insight into the binding mechanisms of the Omicron variants. Of particular interest are the differences that emerged in the BA.4/BA.5 mutational heatmap (Figure 3D). The structure of the BA.4/BA.5 RBD–ACE2 complex is similar to other variants, but this variant features the L452Q/R mutation [86]. Additionally, BA.4 and BA.5 have an additional F486V mutation, along with reversion of R493 back to the wild-type Q493.

The structure of the BA.4/BA.5 RBD–ACE2 complex showed a loss of interactions between F486V and the adjacent ACE2 residues F83 and F28 compared to other variants, while Q493 was shown to have reestablished a hydrogen bond interaction with ACE2 K31 residue, an interaction previously lost with the appearance of the Omicron lineage due to the charge repulsion between R498 and K31 [86]. One prominent study of BA.4/BA.5 binding suggested a potential reduction of favorable interactions and binding due to both F486V and R493Q mutations [29], while another study [87] claimed that the F486V mutation in BA.4/BA.5 spike decreases hACE2 binding activity, but also that BA.4/BA.5 RBD showed higher binding affinity to hACE2 compared with BA.1 and BA.2 spike due to R493Q reversion. The mutational heatmap for the BA.4/BA.5 variant revealed more tolerance in the F486V position, but also smaller changes in the hydrophobic positions L453 and L455 (Figure 3D). Evolutionary studies have also indicated that BQ.1 and BQ.1.1 convergently increase viral fitness by acquiring substitutions at the R346, N460, and K444 residues, leading to increased ACE2 binding affinity during evolution from the BA.5 variant [88]. Consistent with the overall increased stability and binding in BQ.1.1, we found an increased number of binding hotspots (Figure 3E), particularly in residues Y449, Y453, L455, and F456 in the middle region of RBD, as well as at positions V486, N487, Y489, F490, Q493, T500, Y501, and H505. The enhanced affinity of the BQ.1.1 RBD against ACE2 has been linked with N460K, even though N460K is not in direct interface with ACE2 [89]. Our results suggest that N460K can contribute to strengthening of the RBD interaction network, and could rigidify other important sites at the RBD interface, thus increasing the contributions of other notable hotspots in the flexible regions. We argue that the BQ.1.1 mutations N40K, R346T, and K444T may act allosterically by stabilizing the key binding interface segments, despite the fact that these sites themselves are not directly involved in the interaction with ACE2.

**Figure 4:**
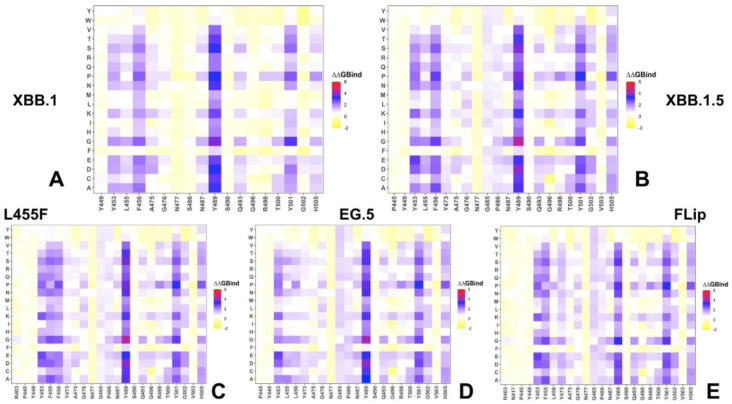
Mutational profiling of the RBD intermolecular interfaces in Omicron RBD–ACE2 complexes. The mutational scanning heatmaps are shown for the interfacial RBD residues in the XBB.1 RBD–ACE2 complex (**A**), XBB.1.5 RBD–ACE2 complex (**B**), XBB.1.5 + L455F RBD–ACE2 complex (**C**), XBB.1.5 + F456L RBD–ACE2 complex (**D**) and XBB.1.5 + L455F/F456L FLip RBD–ACE2 complex (**E**). The standard errors of the mean for binding free energy changes and are within ~0.08–0.18 kcal/mol, using averages based on a total of 1000 samples obtained from the three MD trajectories for each complex.

Mutational scanning heatmaps for the XBB variants highlighted a spectrum of binding free energy changes in the RBD interface residues that maintained stable contacts with the ACE2 receptor during simulations. First, we noticed a consistent presence of shared binding energy hotspots across the XBB RBD–ACE2 complexes that correspond to the hydrophobic centers Y453, L455, F456, Y489, and Y501 (Figure 4). The heatmaps showed that the Y489 and Y501 hotspots are particularly sensitive to modifications across all XBB variants, as most of the substitutions in these positions induce significant destabilizing effects with ΔΔG > 2.0 kcal/mol (Figure 4). Previous DMS experiments have also demonstrated that Y453, L455, F456, F486, and Y489 play a fundamentally critical role in determining both the structural stability of the RBD fold and binding with ACE2 [54,55,56,57,58,59,60]. Mutational maps showed that the increased hydrophobicity in the Y453, L455, and F456 positions, e.g., Y453F/W and L455F/W, may result in neutral or modestly favorable binding changes [89]. Interestingly, the three adjacent RBD hotspots Y453, L455, and F456, located within the central strands comprising the ACE2 interface, showed appreciable epistatic shifts between BA.2 and BQ.1.1 or XBB.1.5, including prominent epistatic changes in the effects of mutations Y453W, L455W/F, and F456L, depending on the genetic background [62].

Mutational heatmaps also showed that mutations in the S486 position of XBB.1 (Figure 4A) are more tolerant as compared to scanning of P486 in the XBB.1.5 variant (Figure 4B), which is consistent with the more favorable ACE2 binding related to S486P modifications. The heatmaps of the XBB.1.5 + F456L (Figure 4D) and XBB.1.5 + L455F/F456L FLip variants (Figure 4E) demonstrated that mutations of P486 at positions other than the reversed P486F modification are typically only moderately destabilizing and are unlikely to impose significant binding loss. These results corroborate DMS experiments that quantified the effects of F486 mutations (F486V/I/S/L/A/P), showing that even though substitutions of F486 can reduce binding, F486P imposes the lowest cost in RBD affinity loss and has the largest increase in RBD expression [54,55,56,60]. Consistent with that, it was found that mutations in this position exemplified by F486V (present in BA.4/BA.5), F486I, F486S (XBB.1), and F486P (shared by XBB.1.5, EG.5, EG.5.1, FLip) represent a convergent evolutionary hotspot, which is one of the major hotspots for escaping neutralization by antibodies. Another critical site of convergent evolution is reversed R493Q mutation, and mutational scanning results showed that modifications at Q493 positions are destabilizing (Figure 4). This is consistent with the notion that R493Q may compensate for partial binding loss incurred by F486P mutation. Our findings are also in agreement with previous studies showing that R493Q reversal may induce an increased affinity of the RBD with ACE2 receptor [90]. The mutational heatmap of the XBB.1.5 Flip RBD residues highlighted the increased stabilization role of the Y453, F455, and L456 residues (Figure 4E).

### 2.3. Probing Epistatic Relationships in the XBB.1.5 and BQ.1.1 Variants Using Mutational Scanning

**Figure 5:**
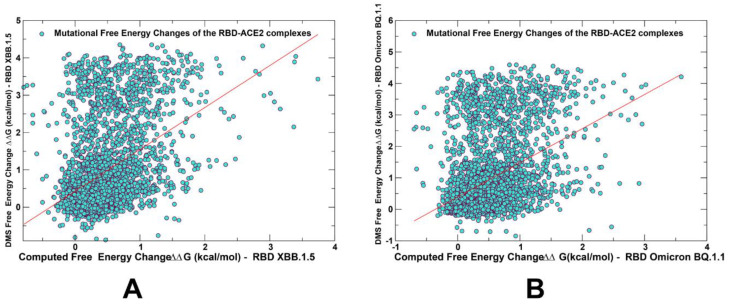
The scatter plots of the DMS-derived binding free energy changes and computational mutational scanning of the RBD residues to estimate mutational effects on ACE2 binding. The effect on ACE2 receptor-binding affinity (Δ log10 *K_D_*) of every single amino-acid mutation in SARS-CoV-2 RBD was experimentally determined by high-throughput titration assays using DMS experiments. The results of computational mutational scanning were averaged over conformational ensembles obtained from all-atom MD simulations. The scatter plot of the experimental and computed binding free energy changes for the Omicron XBB.1.5 RBD–ACE2 complex, pdb id 8WRL (**A**) and BQ.1.1 RBD–ACE2, pdb id 8IF2 8 (**B**). The standard errors of the mean for binding free energy changes are within ~0.16–0.25 kcal/mol, using averages based on a total of 1000 samples obtained from the three MD trajectories for each complex. The red lines on panels (**A**,**B**) represent the regression lines of the corresponding scatter plots.

Recent DMS experiments have examined the impacts of all mutational changes and single-codon deletions in the BQ.1.1 and XBB.1.5 RBDs on ACE2 binding affinity and RBD folding efficiency, revealing the expanded character of epistatic couplings between RBD residues in addition to dramatic epistatic perturbations induced by N501Y, namely prominent epistatic interactions between R493Q reversed mutations and mutations at positions Y453, L455, and F456 that define the newly emerging EG.5.1 and FLip lineages [62]. Strikingly, the epistatic interactions between these sites are background-specific, as mutations Y453W and F456L have been shown to decrease ACE2-binding affinity 2.2- and 6.6-fold in the Omicron BA.2 variant but enhance ACE2-binding affinity 7.1- and 1.9-fold in the XBB.1.5 variant, while L455W has been shown to enhance ACE2-binding affinity 2.5-fold in BA.2, but decrease affinity 7.4-fold in the XBB.1.5 variant [62]. We compared the results of the mutational profiling for XBB.1.5 and BQ.1.1 RBD residues with the recent DMS experiments for three variants (Figure 5) [62].

While correlation between the DMS experiments and mutational scanning data was observed, we also found a significant dispersion of the distributions (Figure 5). It was noticed that the computational predictions of destabilizing changes were often larger than the experimentally observed values. Nonetheless, the scatter plots showed a fairly appreciable correspondence between the predicted and experimental free energy differences for large destabilizing changes with ΔΔG > 2.0 kcal/mol (Figure 5). This allowed for identification of the major binding affinity hotspots, where mutations cause pronounced energetic changes.

To validate the computational model and gain further insights into mechanisms of epistatic couplings of the RBD hotspots, we probed the role of Omicron changes in BA.2 to BQ.1.1 and XBB.1.5 that could be responsible for these sign epistatic shifts. Based on the experimental analysis [46], the residues 453, 455, and 456 were found to be in close proximity to residue 493, which underwent changes during virus evolution from Q493R in the BA.1 and BA.2 variants, and reversed R493Q in BQ.1.1 and XBB.1.5.

**Figure 6:**
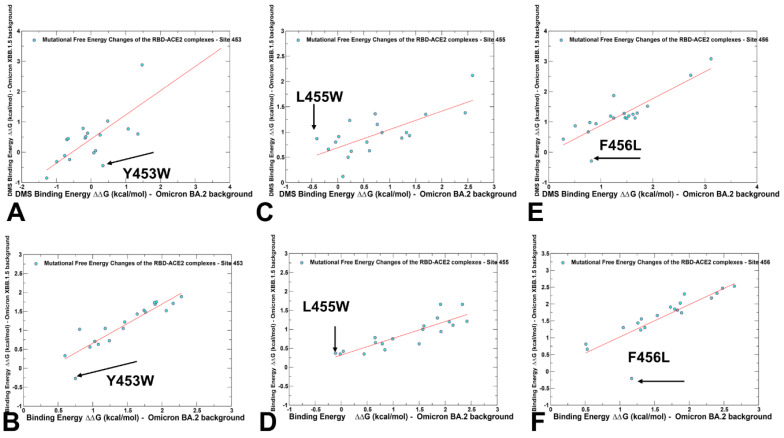
A comparison of the scatter plots for the DMS-based free energy changes and computational mutational scanning of the RBD residues in the different genetic backgrounds. The effect on ACE2 receptor-binding affinity (Δ log10 *K_D_*) of every single amino-acid mutation in SARS-CoV-2 RBD was experimentally determined by DMS experiments. The results of computational mutational scanning were averaged over conformational ensembles obtained from all-atom MD simulations. The standard errors of the mean for binding free energy changes are within ~0.11–0.19 kcal/mol using averages based on a total of 1000 samples obtained from the three MD trajectories for each complex. (**A**) The scatter plot of the experimental DMS free energy changes induced by mutations of Y453 in the BA.2 and XBB.1.5 backgrounds. (**B**) The scatter plot of the computed free energy changes induced by mutations of Y453 in the BA.2 (Q493R) background and XBB.1.5 (R493Q) background. (**C**) The scatter plot of the experimental DMS free energy changes induced by mutations of L455 in the BA.2 and XBB.1.5 backgrounds. (**D**) The scatter plot of the computed free energy changes induced by mutations of L455 in the BA.2 and XBB.1.5 backgrounds. (**E**) The scatter plot of the experimental DMS free energy changes induced by mutations of F456 in the BA.2 and XBB.1.5 backgrounds. (**F**) The scatter plot of the computed free energy changes induced by mutations of F456 in the BA.2 and XBB.1.5 backgrounds. The position of the Y453W, L455W, and F456L mutations are indicated by arrows and annotated. The red lines on panels (**A**,**B**) represent the regression lines of the corresponding scatter plots.

We compared the DMS and computed correlation plots in which binding free energy changes were evaluated in the BA.2 and XBB.1.5 backgrounds for residues Y453, L455, and F456 (Figure 6).

**Figure 7:**
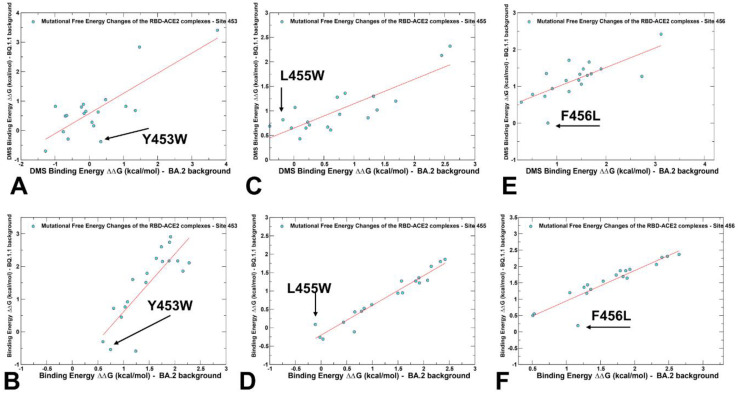
A comparison of the scatter plots for the DMS-based free energy changes and computational mutational scanning of the RBD residues in the different genetic backgrounds. The effect on ACE2 receptor-binding affinity (Δ log10 *K_D_*) of every single amino-acid mutation in SARS-CoV-2 RBD was experimentally determined by DMS experiments. The results of computational mutational scanning were averaged over conformational ensembles obtained from all-atom MD simulations. The standard errors of the mean for binding free energy changes are within ~0.14–0.22 kcal/mol using averages based on a total of 1000 samples obtained from the three MD trajectories for each complex. (**A**) The scatter plot of the experimental DMS free energy changes induced by mutations of Y453 in the BA.2 and BQ.1.1 backgrounds. (**B**) The scatter plot of the computed free energy changes induced by mutations of Y453 in the BA.2 (Q493R background) and BQ.1.1 (R493Q backgrounds). (**C**) The scatter plot of the experimental DMS free energy changes induced by mutations of L455 in the BA.2 and BQ.1.1 backgrounds. (**D**) The scatter plot of the computed free energy changes induced by mutations of L455 in the BA.2 and BQ.1.1 backgrounds. (**E**) The scatter plot of the experimental DMS free energy changes induced by mutations of F456 in the BA.2 and BQ.1.1 backgrounds. (**F**) The scatter plot of the computed free energy changes induced by mutations of F456 in the BA.2 and BQ.1.1 backgrounds. The position of the Y453W, L455W, and F456L mutations are indicated by arrows and annotated. The red lines on panels (**A**,**B**) represent the regression lines of the corresponding scatter plots.

The central focus was on the epistatic changes observed in the DMS data for the Y453W, L455W, and F456L mutations in the BA.2 and XBB.1.5 variants. This analysis allowed us to highlight the differences in the effects of mutations depending on the genetic background, and therefore examine potential epistatic contributions. We found considerable correspondence in patterns of DMS and computed binding free energy changes, strikingly identifying the same critical mutations as outliers from the linear relationships. It should be noted that strong correlations between binding free energy changes induced by mutations in two diverse backgrounds point to positions that produce the same functional effect regardless of the effects of other residues. This would typically result in the additive contribution to the binding free energy. Epistasis is a genetic phenomenon where the effect of one mutation can be altered depending on the presence of other mutations, resulting in non-additive impacts of mutations on specific functions. The pronounced deviations from linear correspondences in these plots can be attributed to mutations which have dramatically different effects in the BA.2 and XBB.1.5 variants, and therefore may be implicated in non-additive epistatic relationships (Figure 6). The scatter plots of binding free energy changes caused by mutations of Y453 in the BA.2 and XBB.1.5 backgrounds showed a linear pattern in both DMS analysis (Figure 6A) and computed value (Figure 6B). The Y453W mutation experimentally yielded ΔG = 0.34 kcal/mol in the BA.2 background, pointing to a destabilization effect and an opposite favorable effect of ΔG = −0.44 kcal/mol in the XBB.1.5 variant (Figure 6A). For the computed changes due to Y453F mutation, the corresponding values were ΔG = 0.75 kcal/mol in the BA.2 variant, and ΔG = −0.27 kcal/mol in XBB.1.5 (Figure 7B).

These results are in excellent agreement with the experiment highlighting epistatic shifts between BA.2 and XBB.1.5, manifested in this case in a dramatic change in the effects of mutations in different variants [62]. Even more dramatic changes in epistatic shifts were evident in DMS analysis of the L455W mutation, where the DMS data yielded a stabilizing effect of ΔG = −0.4 kcal/mol in the BA.2 variant, and an opposite effect of ΔG = 0.87 kcal/mol in the XBB.1.5 variant (Figure 6C). The computational results reproduced the opposite trend of L455W in different backgrounds, but the free energy changes were smaller at ΔG = −0.11 kcal/mol in the BA.2 variant, and ΔG = 0.37 kcal/mol in the XBB.1.5 variant (Figure 6D). According to the DMS analysis, the mutational change at F456L is favorable in the XBB.1.5 variant, causing ΔG = −0.29 kcal/mol stabilization, but is unfavorable in BA.2, inducing a significant destabilizing effect of ΔG = 0.82 kcal/mol (Figure 6E). Our computational predictions were consistent with the experiments, showing that F456L is destabilizing with ΔG = 1.17 kcal/mol in the BA.2 variant, but becomes advantageous for binding in the XBB.1.5 + F456L mutant, leading to ΔG = −0.21 kcal/mol (Figure 6F).

Similar agreements with the DMS experiments were found in mutational scanning of the RBD residues 453, 455, and 456 in the BQ.1.1 variant. The DMS yielded ΔG = 0.34 kcal/mol for Y453W in the BA.2 variant and ΔG = −0.38 kcal/mol in the BQ.1.1 variant (Figure 7A). For the computed changes due to Y453F mutation, the corresponding values were ΔG = 0.75 kcal/mol in BA.2, and ΔG = −0.54 kcal/mol in BQ.1.1 (Figure 7B). For L455W mutation, the DMS data yielded a stabilizing effect of ΔG = −0.4 kcal/mol in the BA.2 variant, and an opposite effect of ΔG = 0.69 kcal/mol in the BQ.1.1 variant (Figure 7C). The computational results displayed ΔG = −0.11 kcal/mol in the BA.2 variant and ΔG = 0.09 kcal/mol in BQ.1.1, indicating a less dramatic shift in the BQ.1.1 variant compared to the experimental evidence (Figure 7D). F456L was found to be favorable in the BQ.1.1 variant, causing ΔG = −0.29 kcal/mol stabilization, while being unfavorable in BA.2, inducing a significant destabilizing effect of ΔG = 0.82 kcal/mol (Figure 7E). According to in silico analysis, F456L was highly unfavorable (ΔG = 1.17 kcal/mol) in the BA.2 variant, but neutral in in the BQ.1.1 (ΔG = 0.19 kcal/mol) (Figure 7F). The results demonstrated that epistatic couplings may be less significant when shifting from BA.2 to BQ.1.1 variants as compared to the stronger nonadditive effect in the XBB.1.5 variant.

The results confirmed the experimentally observed epistatic couplings for the XBB.1.5 and BA.2 variants, where the Y453W and F456L mutations had more favorable effects on binding when coupled with Q493 (XBB.1.5) rather than R493 (BA.2). At the same time, L455W showed a more favorable binding with R493 in the BA.2 background. The F456L mutation is particularly well tolerated in XBB.1.5. indicating strong epistatic contributions are important factors that may drive the evolution of the XBB.1.5 variant. While an extensive epistatic shift was discovered between Q498R and N501Y mutation combinations [57,58], the epistatic interactions between Q493, L455F, and F456L were more gradual. These gradual epistatic changes enable strong ACE2 binding by amplifying contributions of a small number of binding hotspots, while deploying mutations in other positions to combat antibody binding. Recent evolutionary studies have suggested a cumulative effect of many small-effect epistatic modifications, and in the background of gradual epistatic drifts, a few mutations may occasionally undergo substantial changes in their effects [91,92].

### 2.4. Network-Based Community Analysis of Epistatic Couplings in the RBD–ACE2 Complexes

To characterize and rationalize the experimentally observed epistatic effects of the Omicron mutations, we explored a previously introduced simple clique-based network model used for describing the non-additive effects of RBD residues. Using the equilibrium ensembles and dynamic network modeling of the RBD–ACE2 complexes, we applied mutational scanning to perturb modular network organization, represented by a chain of inter-connected sable 3-cliques. Specifically, we calculated the probability by which mutational sites would belong to the same interfacial 3-clique. For this, we generated an ensemble of 1000 protein conformations from MD simulations of the studied RBD–ACE2 complexes. To systematically estimate the non-additive effects of XBB.1.5 mutations and L455F/F456L double-site mutations, we constructed dynamic network structures for each mutant and determined the topological quantities of these networks. By using mutational changes in the Omicron positions over the course of the MD simulation trajectory for the RBD–ACE2 complexes, we attributed RBD and ACE2 interfacial sites that belonged to the same 3-clique to have local non-additive effects, while the effects of specific mutations on changes affected the entire distribution, and the total number of 3-cliques at the RBD–ACE2 interface were attributed to long-range epistatic relationships. If mutational sites are arranged in a 3-clique structure, all three sites are connected to each other. As a result, when one site is mutated, it will have a greater effect on the stability of the complex, because the other two sites will also be affected. Therefore, the presence of a stable 3-clique structure can be used as a first predictor of potential local non-additive effects.

**Figure 8:**
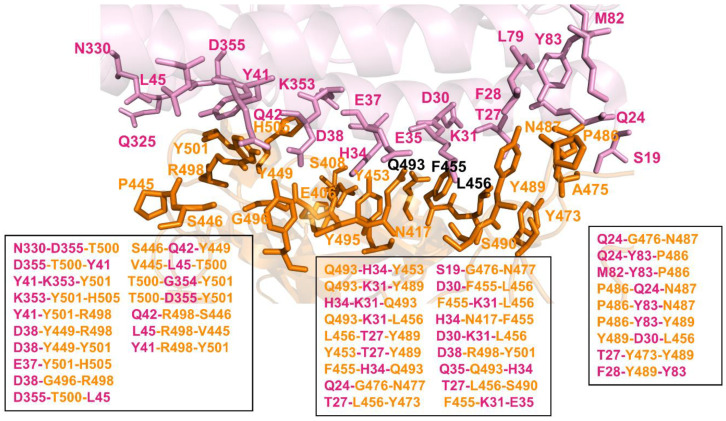
Structural mapping and full annotation of the intermolecular 3-cliques for the XBB.1.5 L455F/F456l FLip RBD–ACE2 complex. The RBD binding interface residues are shown in orange sticks and ACE2 binding interface residues are shown in pink sticks.

We computed and compared the distribution of stability in MD-simulated 3-cliques in the XBB.1.5 FLip RBD–ACE2 complex (Figure 8), showing that the R498 and Y501 sites can promote a larger number of stable 3-cliques at the central interfacial patch. These results highlighted the role of the Q493 position in anchoring multiple interaction clusters with ACE2 residues, while also indicating some level of dynamic coupling with Y453 and Y489 residues. Q493 participates in the following stable cliques: Q493-H34-Y453, Q493-K31-Y489, H34-K31-Q493, Q493-K31-F456L, L455F-H34-Q493, and Q35-Q493-H34. In addition, we detected D30-L455F-F456L, L455F-K31-F456L, F456L-T27-Y489, and D30-K31-F456L cliques that all share a F456L site and link F456L with L455F and D30, K31, and T27 of ACE2 (Figure 8). This network-based community analysis revealed that the R498, Y501, Q493, and F456L positions mediate the vast majority of 3-cliques at the binding interface, and therefore can be responsible for modulating non-additive epistatic relationships between RBD residues.

The results are consistent with the DMS studies which discovered extensive epistasis between the R498 and Y501 combination, also revealing that aromatic substitutions are not tolerated simultaneously at these positions [57,58,62]. The network analysis also revealed strong couplings in 3-cliques between Q493, Y453, L455F, and F456L residues, which are dominated by mediators at the Q493 and F456L positions (Figure 8). According to the community analysis, the non-additive effects may be accentuated by a chain of linked 3-cliques containing Q493 and F456L residues, in which each pair of nodes/residues is connected by an edge, indicating a strong mutual interaction among amino acids on these nodes. These observations showed that the cliques at the middle portion of the interface are mediated through couplings between Q493, L455F, and F456L. Interestingly, the largest number of cliques include Q493 and F456L, suggesting that these residues may be key mediators of non-additive couplings. This also agrees with the latest DMS analysis of XBB variants, showing the ongoing epistatic drift and epistatic interaction between R493Q reversion and mutations at the 453, 455, and 456 positions [62]. The network community analysis showed that the topology of the stable interfacial cliques is preserved between the XBB.1.5 and XBB.1.5 FLip complexes, but the stability of these cliques and their simulation life time can change. To quantify the degree of epistasis, we also calculated the ratio of *P_ab_* after double mutations to *P_ab_* after single mutations. If the probability of two sites belonging to the same 3-clique during simulation increased after double mutations, it would indicate that there was an epistatic effect between the two sites.

We found that the simulation lifetime of stable 3-cliques that involve combined mutations R493Q, L455F, and F456L, particularly for cliques Q493-K31-F456L, L455F-H34-Q493, D30-L455F-F456L, and L455F-K31-F456L, could increase from ~65–70% for XBB.1.5 to 85–90% for these cliques with mutated L455 and F456 in the XBB.1.5 FLip variant complex. Hence, the non-additive interactions within cliques that are mediated by R493Q and F456L may be strengthened, leading to enhanced stability of the binding interface in the FLip RBD–ACE2 complex. Hence, a clique-based network model can identify highly correlated and potentially non-additive RBD sites and distinguish them from other mutational sites that are less likely to experience epistatic shifts. To conclude, mutational profiling combined with network-based community analysis suggested that the Q493, L455, and F456 sites mediate stable communities at the binding interface and serve as mediators of non-additive couplings.

### 2.5. Mutational Profiling of Spike Protein Binding with Monoclonal RBD Antibodies: Revealing Central Role of F456L and F486P Mutations in Immune Evasion

**Figure 9:**
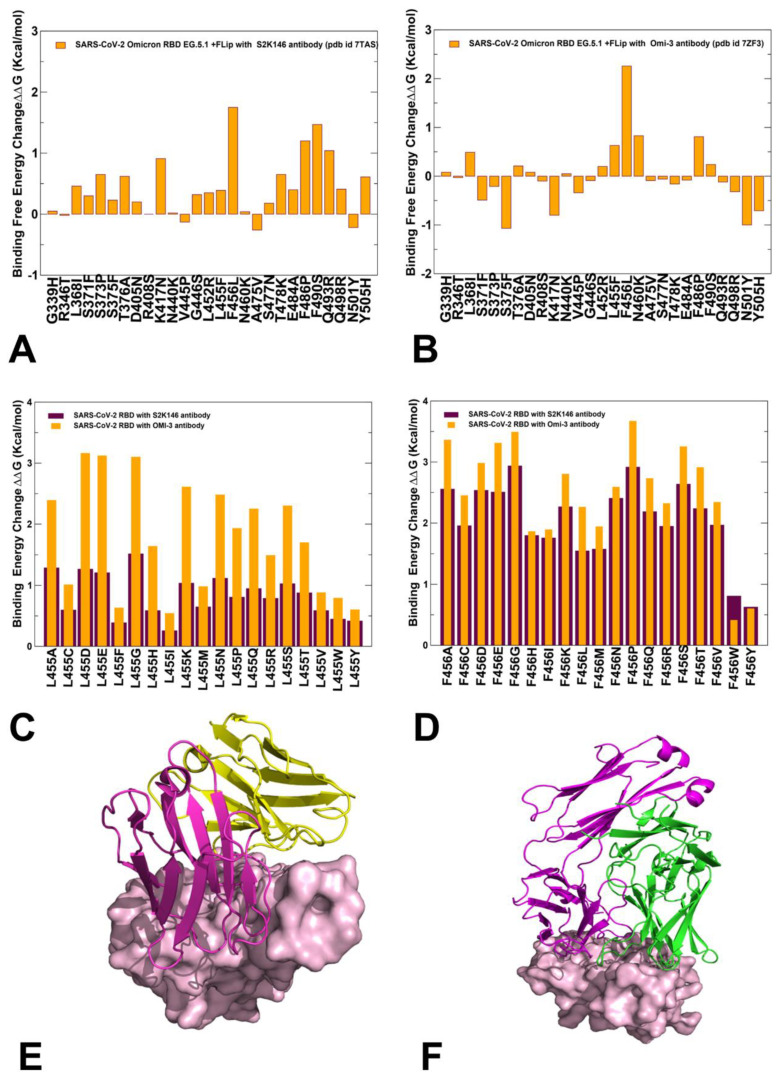
Structure-based mutational profiling of the S-RBD complexes with class 1 of RBD antibodies. The mutational profiling evaluates the binding energy changes induced by XBB.1.5 mutations in the RBD-antibody complexes. Mutational profiling of the S-RBD complex with S2K146 (**A**) and S-RBD Omicron complex with Omi-3 antibody (**B**). The binding free energy changes are shown in orange filled bars. The positive binding free energy values ΔΔG correspond to destabilizing changes and negative binding free energy changes are associated with stabilizing changes. (**C**) Mutational scanning of L455 residue in the RBD-S2K146 complex (maroon bars) and RBD-Omi-3 complex (orange bars). (**D**) Mutational scanning of F456 residue in the RBD-S2K146 complex (maroon bars) and RBD-Omi-3 complex (orange bars). The experimental structures of the RBD-antibody complexes are shown for RBD-S2K146 (**E**), RBD-Omi-3 (**F**). The RBD is shown in pink-colored surface representation and the antibodies are shown in ribbons (heavy chain in magenta and light chain in green-colored ribbons).

We performed structure-based mutational analysis of the S protein binding with different classes of RBD-targeted antibodies, focusing on the role of XBB.1.5 mutations as well as L455F, F456L, and FLip mutations in mediating resistance to class one antibodies. The latest functional studies have shown that the EG.5 and EG.5.1 variants display resistance against S2K146 antibody and a more significant neutralization resistance to Omi-3, Omi-18, Omi-42, and BD-515 class one monoclonal antibodies [42]. For comparison with the experimental data, we specifically examined S2K146 and Omi-3 antibodies, which were experimentally evaluated for evasion properties against the XBB.1.5 + F456L (EG.5), EG.5.1, and XBC.1.6 variants [42,43]. The structures of the RBD-targeted antibodies used in this analysis included S-RBD complex with S2K146 (pdb id 7TAS) [93] and S-RBD Omicron complex with Omi-3 (pdb id 7ZF3) [94]. Mutational profiling analysis of the RBD-antibody complexes for the XBB.1.5, XBB.1.5 + L455F, XBB.1.5 + F456L, and XBB.1.5 Flip variants (Figure 9) allowed for direct comparison, with the reported fold changes in binding constants of antibody binding for these variants in the background of XBB.1.5.

In the analysis of mutational scanning, we specifically focused on the S-antibody binding energy changes induced by XBB.1.5 mutations and L455F and F456L modifications (Figure 9). The binding free energy changes in the S-RBD complex with S2K146 (Figure 9A) showed appreciable losses of binding upon F456L, F486P, and F490S mutation, revealing that these substitutions are deleterious for S2K146 binding. At the same time, L455F induced only modest destabilization changes (Figure 9A). For the Omi-3 antibody, we found that the F456 position presented the dominant binding affinity hotspot, as the destabilization effect of the F456L mutation (ΔΔG = 2.26 kcal/mol) was significantly stronger than that of the S2K146 antibody (Figure 9B). Common to both S2K146 and Omi-3 antibodies, we also observed a considerable loss of binding due to F486P mutation (Figure 9A,B). Specific for Omi-3 antibody was a destabilizing role of N460K mutation, inducing loss of binding with ΔΔG = 0.83 kcal/mol (Figure 9B). To further examine the effects of mutations in the L455 and F456 positions on antibody binding, we also performed complete mutational scanning of these positions against S2K146 (Figure 9C) and Omi-3 (Figure 9D). We observed two interesting trends in this analysis. First, all modifications of L455 and F456 positions resulted in appreciable destabilization changes, and for both positions, the loss of binding was more significant for the Omi-3 antibody. Importantly, we also found that binding losses for both antibodies were markedly larger upon mutations in the F456 position (Figure 9D). Furthermore, while both the L455 and F456 sites correspond to the binding hotspots for Omi-3 antibody, F456 was found to be far more significant than L455 for binding with S2K146. These findings are in excellent agreement with neutralization profiling assays showing that the XBB.1.5 + F456L and EG.5.1 lineages are strongly resistant against Omi-3, Omi-18, and Omi-42 antibodies, but cause less considerable loss of binding for S2K146 antibody [42]. Mutational scanning analysis of the XBB-antibody binding and experimental evidence suggested that rapidly surging EG.5 and EG.5.1 variants may have evolved to improve immune escape against class one RBD antibodies by using F456L and F486P mutations, but this effect does not seem to be uniform, and some monoclonal antibodies, such as S2K146, could still bind fairly strongly with XBB variants [42].

Together, the results of mutational scanning and binding calculations of variants with ACE2 and antibodies clarified the role of FLip mutations in balancing fitness requirements for strong ACE2 binding and robust antibody escape. Indeed, our results suggested that F456L alone may not have a significant effect on ACE2 binding, but could instead mediate strong epistatic couplings with L455F and Q453 to amplify the favorable contributions of these residues in ACE2 interactions. At the same time, F456L together with F486P mutations were shown to be central for mediating antibody resistance, which is consistent with functional experiments showing that the decreased neutralization of EG.5.1 relative to XBB.1.5 is primarily driven by XBB.1.5-F456L mutation [42,43]. The findings of our study provide important quantitative rationale for the latest experimental evidence, which showed that ACE2 binding can be amplified via epistatic interactions of physically proximal binding hotspots, including Y501, R498, Q493, L455, and F456 residues [46,57,58]. Structure-based mutational scanning of the RBD binding interfaces with representative class RBD antibodies characterized the role of the L455F and F456L mutations in eliciting broad resistance to neutralization, confirming that F456L and F486P may function as primary drivers of immune escape while individually incurring moderate changes in ACE2 binding. Our data also pointed to the key role of R493Q mutation in modulating affinity-enhancing effects of mutational changes in Y453, L455, and F456, showing that several key RBD hotspots (N501Y, Q498R and R493Q) can exploit epistatic couplings to enable strong ACE2 binding affinity.

The observed inter-dependence between binding affinity hotspots and antibody resistance substitutions that is manifested by the epistatic couplings of Y501, R498, Q493, L455, and F456 residues can facilitate antibody escape and affect the direction of virus evolution, with potential implications for vaccine design [59]. We suggest that through epistatic couplings revealed in the binding computations and network-based community analysis, the XBB lineages can leverage and amplify the favorable effect of the binding energy hotspots (L455, F45, Y489, Q493, R498, and Y501) on ACE2 binding, while the cumulative contribution of other RBD positions may be balanced against their prominent role in invoking immune evasion. Our findings support a hypothesis according to which the impact on ACE2 binding affinity is mediated through a small group of universal binding hotspots, while the effect of immune evasion could be more variant-dependent and modulated through recruitment of different mutational sites in the adaptable RBD regions [95,96,97,98].

## 3. Materials and Methods

### 3.1. Structure Preparation and Molecular Dynamics Simulations

The crystal and cryo-EM structures of the Omicron RBD–ACE2 complexes were obtained from the Protein Data Bank. For simulated structures, hydrogen atoms and missing residues were initially added and assigned according to the WHATIF program web interface [99]. The missing regions were reconstructed and optimized using the template-based loop prediction approach ArchPRED [100]. The side chain rotamers were refined and optimized using the SCWRL4 tool [101]. The protonation states for all the titratable residues of the ACE2 and RBD proteins were predicted at pH 7.0 using the Propka 3.1 software and web server [102,103]. The protein structures were then optimized using atomic-level energy minimization with composite physics and knowledge-based force fields implemented in the 3Drefine method [104,105]. We considered glycans that were resolved in the structures. A NAMD 2.13-multicore-CUDA package [106] with CHARMM36 force field [107] was employed to perform 1µs all-atom MD simulations for the Omicron RBD–ACE2 complexes. The structures of the SARS-CoV-2 S–RBD complexes were prepared using Visual Molecular Dynamics (VMD 1.9.3) [108], x and with the CHARMM-GUI web server [109,110] using the Solutions Builder tool. Hydrogen atoms were modeled onto the structures prior to solvation with TIP3P water molecules [111] in a periodic box that extended 10 Å beyond any protein atom in the system. To neutralize the biological system before the simulation, Na^+^ and Cl^−^ ions were added in physiological concentrations to achieve charge neutrality, and a salt concentration of 150 mM of NaCl was used to mimic a physiological concentration. All Na^+^ and Cl^−^ ions were placed at least 8 Å away from any protein atoms and from each other. MD simulations were typically performed in an aqueous environment in which the number of ions remained fixed for the duration of the simulation, with a minimally neutralizing ion environment or salt pairs to match the macroscopic salt concentration [112]. All protein systems were subjected to a minimization protocol consisting of two stages. First, minimization was performed for 100,000 steps with all the hydrogen-containing bonds constrained and the protein atoms fixed. In the second stage, minimization was performed for 50,000 steps with all the protein backbone atoms fixed, and for an additional 10,000 steps with no fixed atoms. After minimization, the protein systems were equilibrated in steps by gradually increasing the system temperature in steps of 20 K, increasing from 10 K to 310 K, and at each step, a 1ns equilibration was performed, maintaining a restraint of 10 kcal mol^−1^ Å^−2^ on the protein C_α_ atoms. After the restraints on the protein atoms were removed, the system was equilibrated for an additional 10 ns. Long-range, non-bonded van der Waals interactions were computed using an atom-based cutoff of 12 Å, with the switching function beginning at 10 Å and reaching zero at 14 Å. The SHAKE method was used to constrain all the bonds associated with hydrogen atoms. The simulations were run using a leap-frog integrator with a 2 fs integration time step. The ShakeH algorithm in NAMD was applied for the water molecule constraints. The long-range electrostatic interactions were calculated using the particle mesh Ewald method [113] with a cut-off of 1.0 nm and a fourth-order (cubic) interpolation. The simulations were performed under an NPT ensemble with a Langevin thermostat and a Nosé–Hoover Langevin piston at 310 K and 1 atm. The damping coefficient (gamma) of the Langevin thermostat was 1/ps. In NAMD, the Nosé–Hoover Langevin piston method is a combination of the Nosé–Hoover constant pressure method [114] and piston fluctuation control implemented using Langevin dynamics [115,116]. An NPT production simulation was run on equilibrated structures for 1µs, keeping a constant pressure (1 atm) and the temperature at 310 K.

### 3.2. Mutational Scanning and Binding Free Energy Computations

Mutational scanning analysis of the binding epitope residues was conducted for all of the studied RBD–ACE2 complexes. Each binding epitope residue was systematically mutated using all substitutions and corresponding binding free energy changes were computed using the BeAtMuSiC approach and webserver [117]. BeAtMuSiC is based on statistical potentials describing the pairwise inter-residue distances, backbone torsion angles, and solvent accessibilities, and considers the effect of the mutation on the strength of the interactions at the interface and on the overall stability of the complex. The binding free energy of the protein–protein complex can be expressed as the difference in the folding free energy of the complex and the folding free energies of the two protein binding partners:

(1) $$\Delta G_{bind}=G^{com}-G^{A}-G^{B}$$

The change of the binding energy due to a mutation was then calculated as the following:

(2) $$\Delta \Delta G_{bind}=\Delta G_{bind}^{mut}-\Delta G_{bind}^{wt}$$

We computed the ensemble-averaged binding free energy changes using equilibrium samples from simulation trajectories. The binding free energy changes were computed by averaging the results of over 1000 equilibrium samples for each of the studied systems.

The BeAtMuSiC approach is comparable to other knowledge-based structural methods such as Dcomplex [118] and physics-based FoldX potentials [119]. A large-scale in silico mutagenesis study using the BeAtMuSiC approach profiled all possible point mutations in the RBD residues based on their stability and binding with antibodies and the ACE2 receptor, showing that predictions agreed well with various experimental, epidemiological, and clinical data [120].

### 3.3. Network-Based Community Analysis and Clique-Based Model of Epistatic Interactions

A graph-based representation of protein structures [121] was used to represent residues as network nodes and the inter-residue edges were used to describe non-covalent residue interactions. The network edges that defined residue connectivity were based on non-covalent interactions between residue side-chains. The weights of the network edges in the residue interaction networks were determined by dynamic residue cross-correlations obtained from MD simulations [122] and coevolutionary couplings between residues measured by mutual information scores [123]. The edge lengths in the network were obtained using the generalized correlation coefficients associated with the dynamic correlation and mutual information shared by each pair of residues. The analysis of the interaction networks was performed using network parameters such as cliques and communities. The Girvan–Newman algorithm [124] was used to identify local communities. In this approach, edge centrality (also termed edge betweenness) was defined as the ratio of all the shortest paths passing through a particular edge to the total number of shortest paths in the network. The method employed an iterative elimination of edges with the highest number of the shortest paths that go through them. By eliminating edges, the network broke down into smaller communities. The algorithm started with one vertex, calculated edge weights for paths going through that vertex, and then repeated it for every vertex in the graph, summing the weights for every edge. However, in complex and dynamic protein structure networks, the number of edges often has the same highest edge betweenness. An improvement on the Girvan–Newman method was implemented, and the algorithmic details of this modified scheme were given in our recent studies [125].

The k-cliques are complete sub graphs of size k in which each node is connected to every other node. A k-clique community is determined by the clique percolation method [126], a subgraph containing k-cliques that can be reached from each other through a series of adjacent *k*-cliques. We used a community definition, which specifies that in a k-clique community, two k-cliques share k−1 or k−2 nodes. Computation of the network parameters was performed using the clique percolation method as implemented in the CFinder program [127]. Given the chosen interaction cutoff $I_{min}$, we typically obtained communities formed as a union of k = 3 and k = 4 cliques. We assumed that residues that belonged to the same clique during simulations would have stronger dynamic and energetic couplings, leading to synchronization and potentially epistatic effects. To examine the epistatic effect of a mutational site, we compared changes in the k-clique community distributions induced by single and double mutations, and calculated the probability by which the two mutational sites belonged to the same interfacial 3-clique [128].

We computed the proportion *P_ab_* of snapshots in the ensemble in which the two mutational sites (*a*, *b*) belonged to the interfacial 3-clique:

(3) $$P_{ab}=\frac{\sum_{i=1}^{N}C_{ab}(i)}{N}$$

$C_{ab}\left(i\right)=$ 1 if (*a*, *b*) belonged to the same 3-clique. *P_ab_* measured the probability that two sites (*a*, *b*) were kept in some 3-clique due to either direct or indirect interactions. The closer *P_ab_* was to 1, the more likely *a* and *b* tended to have a tight connection and potential local epistasis. To further investigate the effect of mutations on the 3-clique probability, we compared changes in *P_ab_* after single and double mutations. If double mutations had a greater effect on *P_ab_* than single mutations, this indicated the potential presence of an epistatic effect between the two sites.

## 4. Conclusions

The results of this study provide a molecular underpinning for experimental findings suggesting that acquiring functionally balanced substitutions, which optimize multiple fitness tradeoffs such as immune evasion, high ACE2 affinity, and adequate conformational adaptability, may represent a prevalent strategy in viral evolutionary trajectory. Functional investigations have revealed that the pathways of evolution leading to significant enhancements in the binding affinity of Omicron RBD variants with hACE2 are relatively limited and demand balancing of diverse fitness tradeoffs, such as maintaining RBD stability, ACE2 binding, and immune evasion. These considerations may limit the scope for the virus to develop mutations that substantially increase ACE2 binding affinity without compromising immune evasion and stability. Consequently, there is growing recognition that evolutionary pressures involve a complex interplay of thermodynamic factors, resulting in the identification of specific Omicron mutational hotspots that enhance ACE2 binding affinity, while allowing other Omicron sites to evolve immune escape capabilities with minimal destabilizing consequences.

The results of this study also have implications for predicting the evolution of Omicron variants through better understanding of how epistasis and allosteric interactions are modulated by mutations. The advent of DMS technologies, ancestral reconstruction techniques, and high-throughput assays for protein function can enable direct assessment of mutational effects. Combining a dynamic view of allostery and exploiting conformational ensembles of S proteins with profiling the effects of individual mutations on binding in different genetic backgrounds can complement DMS experiments and also provide a structural basis for understanding the functional consequences of epistasis. Recent DMS studies [54,55,56,57,58,59,60] have emphasized that mutational effects at different sites in the RBD drift randomly during evolution. These studies and our computational results have indicated that some Omicron RBD mutations can have conserved binding and stability effects across different genetic backgrounds, while the effects of mutations in other RBD positions may vary drastically as a result of epistatic couplings, thus making the effects of mutations less predictable as proteins evolve [92]. The results of our study are consistent with the idea that epistatic couplings between binding affinity hotspots may allow for accumulation of diverse and functionally beneficial (but binding neutral) mutations in other positions to balance immune evasion and ACE2 binding. The revealed patterns are reminiscent of direct evolution studies, showing that enhanced protein stability can promote broader evolvability and emergence of beneficial mutations through suppression of deleterious changes [96,97,98]. The related ideas of the “epistatic ratchets” and “pivot mutations” that interact with other mutations via epistasis suggest that the formation of multiple epistatically interacting mutations during evolution makes the reversal of ancestral phenotypes increasingly difficult [129,130]. On other hand, phenotypic shifts caused by single or additive genetic changes are likely to be readily reversible. These arguments are consistent with the decisive role of the stable epistatic hotspots Y501 and R498 in ACE2 binding and the presence of epistatic drift in other RBD sites that mediate immune evasion properties and may determine future “pockets” of evolutionary changes. Most recent studies have identified “evolvability-enhancing” mutations that could create a genetic background where subsequent mutations are more likely to be beneficial relative to mutations acquired on an ancestral background [131,132]. The relationships between epistatic interactions and evolution are complex and while the effects of some mutations follow predictable fitness-correlated patterns, these patterns depend on the biological system and on the physical underpinnings of particular phenotypes, such as protein binding. Although the proposed ensemble-based mutational profiling of the spike residues can enable robust assessment of mutational effects on stability and binding with antibodies and the ACE2 receptor, the predictions are limited to a simplified contact-based analysis of epistatic couplings and are often insufficient for capturing complex fitness landscapes of virus evolution. Future studies dissecting mechanisms of Omicron mutations could benefit from the integration of DMS experiments with evolutionary modeling of epistasis, prediction of allosteric interactions, and in silico mutational profiling of conformational ensembles that can quantify the dynamic effect of mutations on allosteric and epistatic interactions across different genetic backgrounds.

## Figures and Tables

**Table 1:** A summary of accumulated mutations in the Omicron variants.

Omicron Variant	Mutational Landscape
BA.1	A67, T95I, G339D, S371L, S373P, S375F, K417N, N440K, G446S, S477N, T478K, E484A, Q493R, G496S, Q498R, N501Y, Y505H, T547K, D614G, H655Y, N679K, P681H, N764K, D796Y, N856K, Q954H, N969K, L981F
BA.2	T19I, G142D, V213G, G339D, S371F, S373P, S375F, T376A, D405N, R408S, K417N, N440K, S477N, T478K, E484A, Q493R, Q498R, N501Y, Y505H, D614G, H655Y, N679K, P681H, N764K, D796Y, Q954H, N969K
BA.4	T19I, G142D, V213G, G339D, S371F, S373P, S375F, T376A, D405N, R408S, K417N, N440K, L452R, S477N, T478K, E484A, F486V, R493Q reversal, Q498R, N501Y, Y505H, D614G, H655Y, N679K, P681H, N764K, D796Y, Q954H, N969K
BA.5	T19I, LPPA24-27S, Del 69-70, G142D, V213G, G339D, S371F, S373P, S375F, T376A, D405N, R408S, K417N, N440K, L452R, S477N, T478K, E484A, F486V, R493Q reversal, Q498R, N501Y, Y505H, D614G, H655Y, N679K, P681H, N764K, D796Y, Q954H, N969K
BQ.1.1	T19I, LPPA24-27S, H69del, V70del, V213G, G142D, G339D, S371F, S373P, S375F, T376A, D405N, R408S, K417N, N440K, K444T, L452R, N460K, S477N, T478K, E484A, F486V, R493Q reversal, Q498R, N501Y, Y505H, D614G, H655Y, N679K, P681H, N764K, D796Y, Q954H, N969K
XBB.1	T19I, V83A, G142D, Del144, H146Q, Q183E, V213E, G252V, G339H, R346T, L368I, S371F, S373P, S375F, T376A, D405N, R408S, K417N, N440K, V445P, G446S, N460K, S477N, T478K, E484A, F486S, F490S, R493Q reversal, Q498R, N501Y, Y505H, D614G, H655Y, N679K, P681H, N764K, D796Y, Q954H, N969K
XBB.1.5	T19I, V83A, G142D, Del144, H146Q, Q183E, V213E, G252V, G339H, R346T, L368I, S371F, S373P, S375F, T376A, D405N, R408S, K417N, N440K, V445P, G446S, N460K, S477N, T478K, E484A, F486P, F490S, R493Q reversal, Q498R, N501Y, Y505H, D614G, H655Y, N679K, P681H, N764K, D796Y, Q954H, N969K
XBB.1.5 + F456L	T19I, V83A, G142D, Del144, H146Q, Q183E, V213E, G252V, G339H, R346T, L368I, S371F, S373P, S375F, T376A, D405N, R408S, K417N, N440K, V445P, G446S, F456L, N460K, S477N, T478K, E484A, F486P, F490S, R493Q reversal, Q498R, N501Y, Y505H, D614G, H655Y, N679K, P681H, N764K, D796Y, Q954H, N969K
XBB.1.5 + L455F/F456L	T19I, V83A, G142D, Del144, H146Q, Q183E, V213E, G252V, G339H, R346T, L368I, S371F, S373P, S375F, T376A, D405N, R408S, K417N, N440K, V445P, G446S, L455F, F456L, N460K, S477N, T478K, E484A, F486P, F490S, R493Q reversal, Q498R, N501Y, Y505H, D614G, H655Y, N679K, P681H, N764K, D796Y, Q954H, N969K

## Data Availability

Data is fully contained within the article. Crystal structures were obtained and downloaded from the Protein Data Bank (http://www.rcsb.org, accessed on 6 February 2024). All simulations were performed using NAMD 2.13 package that was obtained from website https://www.ks.uiuc.edu/Development/Download/, accessed on 1 February 2024. All simulations were performed using the all-atom additive CHARMM36 protein force field that can be obtained from http://mackerell.umaryland.edu/charmm_ff.shtml, accessed on 2 February 2024. The residue interaction network files were obtained for all structures using the Residue Interaction Network Generator (RING) program RING v2.0.1 freely available at http://old.protein.bio.unipd.it/ring/, accessed on 5 February 2024. The computations of network parameters were performed using NAPS program available at https://bioinf.iiit.ac.in/NAPS/index.php, accessed on 10 February 2024 and Cytoscape 3.8.2 environment available at https://cytoscape.org/download.html, accessed on 12 February 2024. The rendering of protein structures was performed using the interactive visualization program UCSF with the ChimeraX package (https://www.rbvi.ucsf.edu/chimerax/, accessed on 15 February 2024) and Pymol (https://pymol.org/2/, accessed on 11 February 2024).

## Supplementary Materials

The following supporting information can be downloaded at: https://www.mdpi.com/article/10.3390/ijms25084281/s1.
