# Supplementary material for: Ensemble-Based Mutational Profiling and Network Analysis of the SARS-CoV-2 Spike Omicron XBB Lineages for Interactions with the ACE2 Receptor and Antibodies: Cooperation of Binding Hotspots in Mediating Epistatic Couplings Underlies Binding Mechanism and Immune Escape
Source: Int J Mol Sci. 2024 Apr 12;25(8):4281. doi: 10.3390/ijms25084281 (PMC11049863; doi:10.3390/ijms25084281)
Supplement: Supplementary file 1 [file ijms-25-04281-s001.zip › ijms-2937550-supplementary.pdf]

Supplementary Materials

Ensemble-Based Mutational Profiling and Network Analysis of the SARS-CoV-2 Spike Omicron XBB Lineages for Interactions with the ACE2 Receptor and Antibodies: Cooperation of Binding Hotspots in Mediating Epistatic Couplings Underlies Binding Mechanism and Immune Escape

Nishank Raisinghani [1], Mohammed Alshahrani [1], Grace Gupta [1] and Gennady Verkhivker [1,2,*]

1 Keck Center for Science and Engineering, Graduate Program in Computational and Data Sciences, Schmid College of Science and Technology, Chapman University, Orange, CA 92866, USA
2 Department of Biomedical and Pharmaceutical Sciences, Chapman University School of Pharmacy, Irvine, CA 92618, USA
* Correspondence: verkhivk@chapman.edu; Tel.: +1-714-516-4586

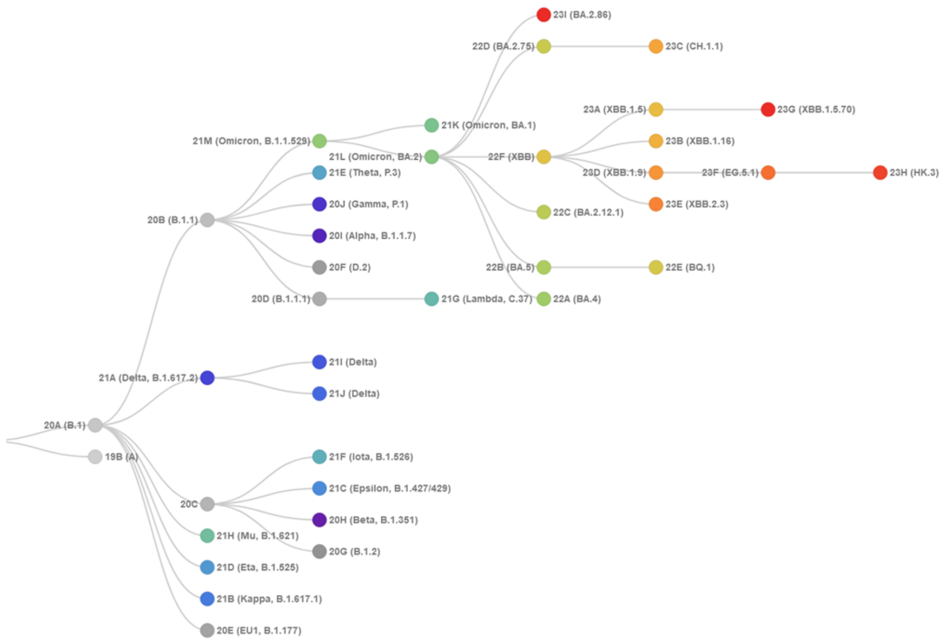

Figure S1. The evolutionary tree of current SARS-CoV-2 clades. XBB.1, XBB.1.5 and XBB.1.5.70 variants are shown on the current tree. The graph is generated using Nextstrain, an open-source project for real time tracking of evolving pathogen populations (https://nextstrain.org/) [39]. The clade 22F corresponds to XBB, 23A corresponds to XBB.1.5, and 23G corresponds to XBB.1.5.70 (XBB.1.5+L455F+F456L) variant. The evolutionary tree highlights that XBB.1.5.70 is a descendant of XBB.1 and XBB.1.5 variants.

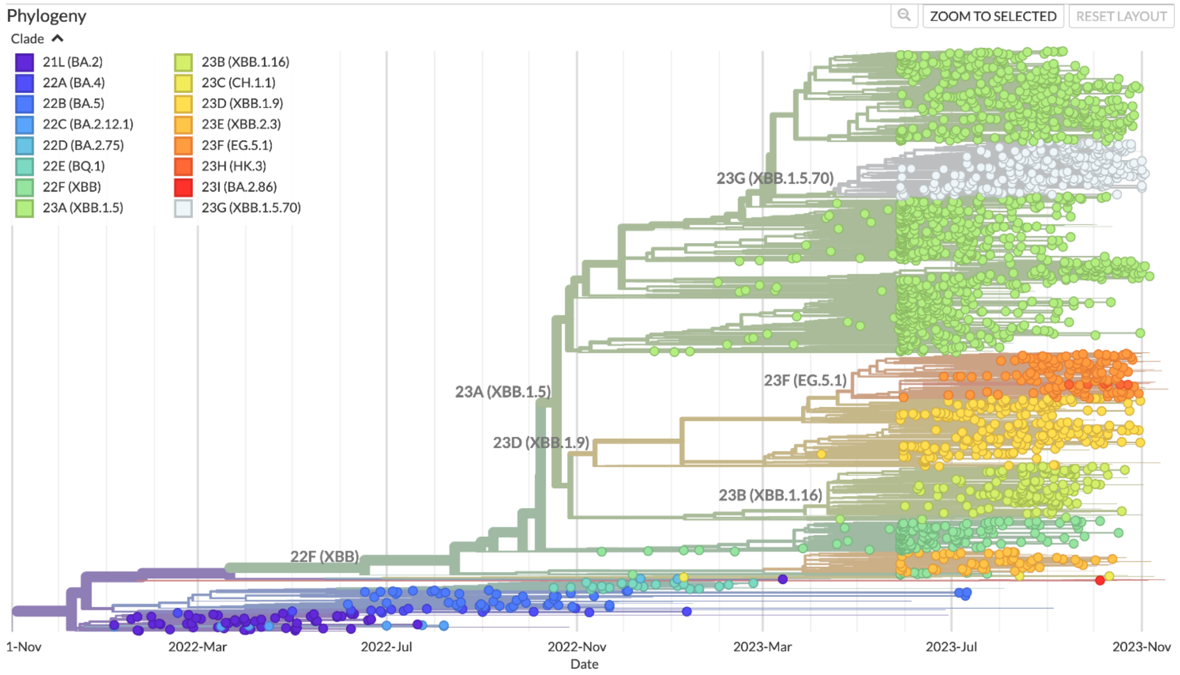

Figure S2. Highlight of the Omicron clade (GSAID Clade 23G) in the time-scaled phylogenetic tree of a representative global subsample between November 2022 and November 2023. Phylogeny has been reconstructed using nextstrain/ncov (https://github.com/nextstrain/ncov.

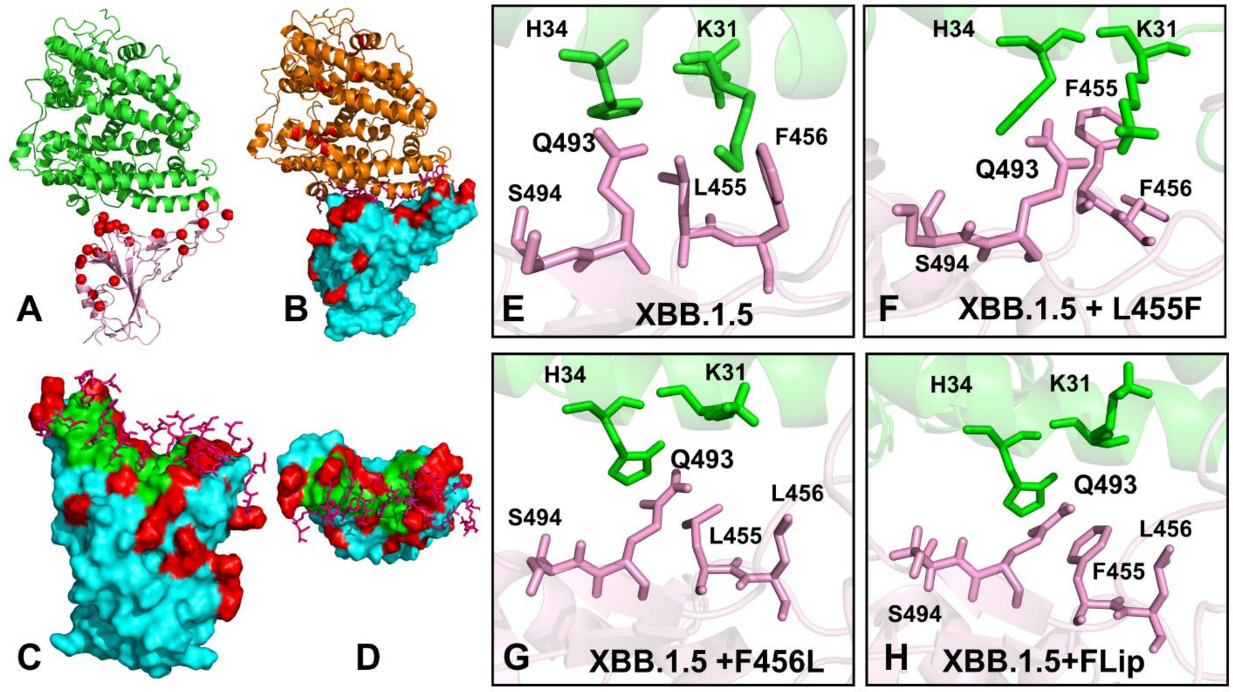

Figure S3. Structural overview of the SARS-CoV-2 S-RBD-ACE2 complexes for Omicron XBB lineages. (A) The cryo-EM structure of the XBB.1.5 RBD-ACE2 complex (pdb id 8WRL). The RBD is shown in pink ribbons, the ACE2 receptor is in green ribbons. The XBB.1.5 RBD mutational sites G339H, R346T, L368I, S371F, S373P, S375F, T376A, D405N, R408S, K417N, N440K, V445P, G446S, N460K, S477N, T478K, E484A, F486P, F490S, R493Q , Q498R, N501Y,

Y505H are shown in red spheres. (B) The surface-based representation of XBB.1.5 RBD-ACE2 complex. The RBD is pink surface, ACE2 is in orange ribbons. The XBB.1.5 mutational positions are in red. (C,D) The front and top view of the S-RBD binding epitope for the XBB.1.5 RBD-ACE2 complex. The RBD is in cyan surface, The binding epitope is in green surface, XBB.1/XBB.1.5 Omicron mutations are in red. The ACE2 binding interface residues are shown in sticks. (E) A closeup of the binding interface residues Q493, L455 and F456 in the cryo-EM structure of the XBB.1.5 RBD-ACE2 complex. The RBD residues are in pink sticks, the ACE2 residues H3 and K31 are in green sticks. (F) A closeup of the binding interface residues Q493, L455F and F456 in the AF2-predicted best model of the XBB.1.5+L455F complex with ACE2. (G) A closeup of the binding interface residues Q493, L455 and F456L in the AF2-predicted best model of the XBB.1.5+F456L complex with ACE2. (H) A closeup of the binding interface residues Q493, L455F and F456L in the AF2-predicted best model of the XBB.1.5+ FLip complex with ACE2. The binding interface residues are shown in pink sticks for RBD sites and green sticks for ACE2 sites. The RBD interface residues Q493, L455F and F456L undergo noticeable rearrangements in the XBB.1.5+FLip complex.

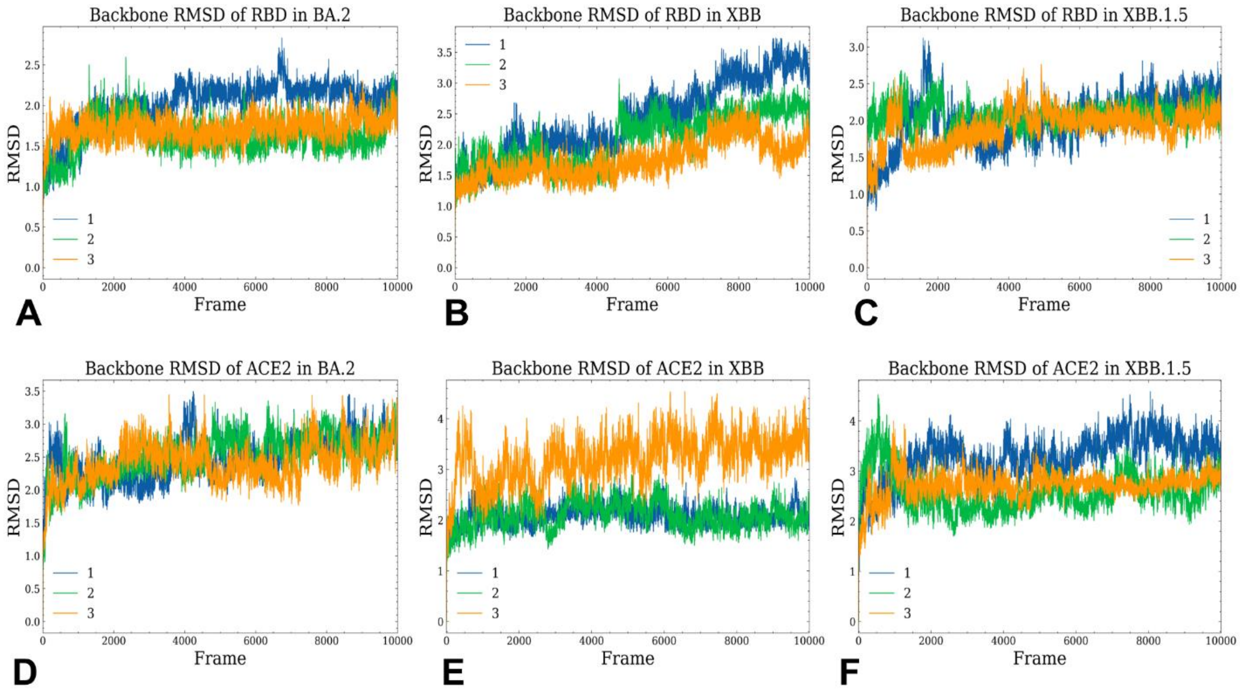

Figure S4. Conformational dynamics profiles obtained from 3 microsecond-long all-atom MD simulations of the Omicron RBD BA.2, XBB.1 and XBB.1.5 complexes with hACE2. The RMSD profiles for the RBD residues obtained from 3 microsecond MD simulations of the Omicron RBD BA.2-ACE2 complex, pdb id 7XB0 (A), Omicron RBD XBB.1-ACE2 complex, pdb id 8IOV (B), and Omicron RBD XBB.1.5-ACE2 complex, pdb id 8WRL (C). The RMSD profiles for ACE2 residues for the Omicron RBD BA.2-ACE2 complex (D), Omicron RBD XBB.1-ACE2 complex (E), and Omicron RBD XBB.1.5-ACE2 complex (F).

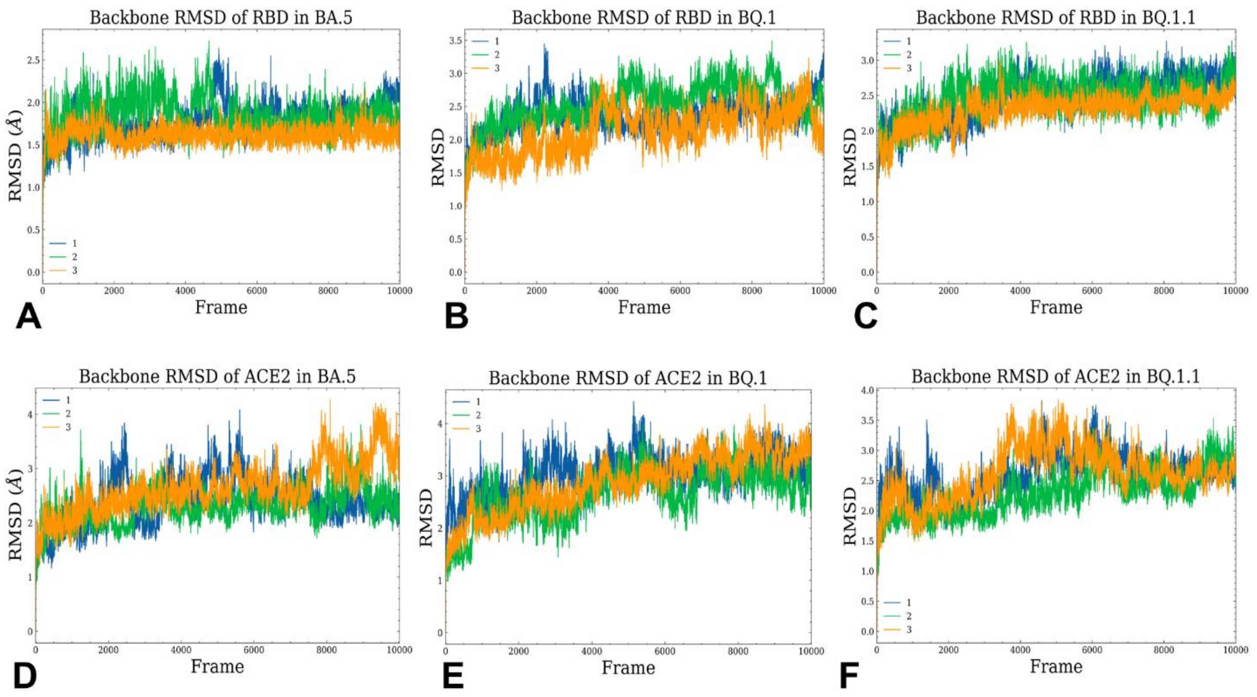

Figure S5. Conformational dynamics profiles obtained from 3 microsecond-long all-atom MD simulations of the Omicron RBD BA.5, BQ.1 and BQ.1.1 complexes with hACE2. The RMSD profiles for the RBD residues obtained from 3 microsecond MD simulations of the Omicron RBD BA.4/BA.5-ACE2 complex, pdb id 8AQS (A), Omicron RBD BQ.1-ACE2 complex (B), and Omicron RBD BQ.1.1-ACE2 complex, pdb id 8IF2 (C). The RMSD profiles for ACE2 residues for the Omicron RBD BA.4/BA.5-ACE2 complex (D), Omicron RBD BQ.1-ACE2 complex (E), and Omicron RBD BQ.1.1-ACE2 complex (F).
